# Host Microbe Interactions in the Lactating Mammary Gland

**DOI:** 10.3389/fmicb.2019.01863

**Published:** 2019-08-13

**Authors:** Olga Sakwinska, Nabil Bosco

**Affiliations:** ^1^Nestlé Research, Nestlé Institute of Health Sciences, Lausanne, Switzerland; ^2^Nestlé Research Singapore Hub, Singapore, Singapore

**Keywords:** human milk, mucosal surface, microbiome, lactation, breastfeeding, mastitis

## Abstract

The bacteria present in human milk constitute the human milk microbiome (hMM). Both the older culture-based work and the more recent studies using molecular detection of bacterial DNA have reached similar conclusions: the hMM mostly consists of commensal staphylococci such as *Staphylococcus epidermidis*, and streptococci. The prevalence of other bacterial groups such lactobacilli varies widely, while the abundance and prevalence of bifidobacteria is generally low. Recently, the hMM became accepted as a part of a physiologically normal state with suggested potential health benefits. Most research on the hMM has focused on its composition and potential effect on the breastfed infant. A major role as a microbiome inoculum for the infant gut has been proposed, but remains to be clearly demonstrated. Herein, we also discuss the emerging connection between the hMM and mammary gland physiology and lactation. Similarities between the mammary gland and mucosal interfaces are considerable, and in particular mucosal-like immune attributes of mammary gland. The potential role of hMM-host interactions in the mammary gland in maternal health is explored with a primary focus on lactational mastitis.

## Introduction

Historically, medicine considered bacteria as dangerous pathogens threatening human health. Only in the last decade came the awareness that humans are colonized in different parts of their bodies by diverse communities of microorganisms collectively called microbiomes. In parallel, there has been increasing recognition that microbiomes play a crucial role in human health through multiple aspects of their metabolic activity and interactions with their host’s physiology. The microbes present in human milk (HM) are no exception. Although their presence in HM has been documented for decades (Carroll et al., 1979), only recently has it become accepted as not only representing a normal physiological state but also as potentially beneficial to the lactating infant. Most research on the human milk microbiome (hMM) focused on its potential effect on the breastfed infant, and particularly on the possibility of bacterial strain transfer to seed colonization of the infant gut. Surprisingly, only few links were made with other research fields where the presence of bacteria in milk is of key importance for the mother.

In this review, we first briefly comment on the recent studies describing the hMM and the evidence for its role in infant and maternal health. Secondly, we explore the newly identified aspects of mammary gland physiology and lactation in connection with the hMM. Further, we discuss the host-microbiome interaction in the mammary gland and its potential effects on maternal health with a primary focus on lactational mastitis.

## Human Milk Microbes Assessed by Culture and Molecular Detection – Findings and Challenges

Live bacteria detected by culture are found in human milk, ranging from 10^1^ to 10^7^ colony forming units (CFU) per mL (Heikkilä and Saris, 2003; Jiménez et al., 2008; Kvist et al., 2008; Martín et al., 2012; Jost et al., 2013; Soto et al., 2014; Soeorg et al., 2017). In comparison, this number of bacteria in any other body fluids such as urine and blood is considered abnormal. Commensal staphylococci (coagulase negative staphylococci, CoNS) and streptococci are consistently reported as predominant taxa in HM. More precise identification revealed *Staphylococcus epidermidis* as the dominant taxon present in 80–100% of samples (Heikkilä and Saris, 2003; Jiménez et al., 2008; Collado et al., 2012; Jost et al., 2013; Soto et al., 2014; Soeorg et al., 2017). Other staphylococci included *S. hominis*, *S. haemolyticus*, and *S. lugdunensis*, and *Streptococcus salivarius* and *S. mitis* among streptococci. The detection of lactobacilli was very variable ranging from less than 3–80%. The main taxa reported were *Lactobacillus plantarum*, *L. fermentum*, *L. casei*, and *L. gasseri* (Albesharat et al., 2011; Martín et al., 2012; Soto et al., 2014). Similar observations were made using samples obtained from milk banks, although the data are rarely collected separately for individual donors (e.g., Almutawif et al., 2017). Many of the taxa found in HM are also found on skin. The differences in collection methods among studies likely explain variability of results. In some studies specific effort was made to collect under aseptic conditions with elimination of the first few microliters to milliliters of milk, with the idea to eliminate what was considered skin contaminants. Others considered that the totality of the bacteria ingested by the infant during breastfeeding is more relevant, therefore the entire sample was collected.

Determining the complete composition of microbiota by culture is laborious and limited to the taxa capable of growth in laboratory conditions. In line with a deluge of studies of the human microbiome using culture independent methods, a majority of recent studies on the hMM used technologies relying on the detection and sequencing of bacterial DNA, targeting specific taxa by quantitative PCR (qPCR), and more recently, amplicon sequencing of the 16S rRNA gene fragments and shotgun metagenomics (reviewed in Fitzstevens et al., 2017 and Table 1). Culture-independent methods relying on DNA detection allow working on frozen samples and permit higher sample throughput. Moreover, sequencing of the 16S rRNA and shotgun metagenomics can be used to detect bacterial taxa difficult to culture. However, sequencing-based technologies in parallel with unprecedented opportunities, represent important challenges, which are particularly pronounced in studies of samples with low bacterial biomass, such as human milk (Salter et al., 2014; Kim et al., 2017). Low levels of bacterial DNA are present in commercial reagent kits and laboratory environment. As the DNA detection methods are typically highly sensitive, even a very small amount of bacterial DNA may produce a signal (Bushman, 2019). In most studies of human milk adequate controls have either not been used or have not been reported, leaving high degree of uncertainty regarding the actual composition of the milk samples (see Table 1). This is further compounded by lack of reporting of the absolute quantity of bacterial DNA or bacterial load, a parameter which is key for biological interpretation. Of note, total DNA measurements are not informative; indeed carefully conducted studies reported that most DNA extracted from hMM samples is of human origin (Ferretti et al., 2018). Sequencing of the 16S rRNA gene is generally informative only at the genus level and thus the identification of bacterial species is usually precluded.

**TABLE 1:** Studies with characterization of bacteria present in human milk by microbiota profiling published since the review of Fitzstevens et al. (2017).

**Predominant taxa**	**Characterization method, sequencing region of 16S rRNA gene**	***N* (subjects)**	**Country**	**Use of negative controls**	**References**
*Streptococcus Staphylococcus*	16S, V4	10	Mexico	Not reported	Dave et al., 2016
*Staphylococcus Streptococcus Pseudomonas*	16S, V4	80	China South Africa Finland Spain	Use of no template control (NTC) reported	Kumar et al., 2016
*Streptococcus Staphylococcus Acinetobacter*	16S, V4	60	China	Not reported	Sakwinska et al., 2016
*Staphylococcaceae Streptococcaceae*	16S, V3–V4	36	Italy	Not reported	Biagi et al., 2017
*Streptococcaceae Pseudomonadaceae Staphylococcaceae*	16S, V1–V2	133	China Taiwan	Not reported	Li et al., 2017
*Pseudomonas Streptococcus Staphylococcus*	16S, V3–V4	10	Ireland	Not reported	Murphy et al., 2017
*Moraxellaceae Streptococcaceae Staphylococcaceae*	16S, V3–V4	107	United States	Use of reagent and amplification controls reported	Pannaraj et al., 2017
*Staphylococcus Streptococcus*	16S, V2, V4	29	Italy	Not reported	Toscano et al., 2017
*Streptococcus Staphylococcus*	16S, V1–V3	21	United States	Use of reagent and amplification controls reported	Williams et al., 2017
*Streptococcus Staphylococcus*	Metagenomics	16	Finland	Not reported	Pärnänen et al., 2018
*Streptococcus Ralstonia Staphylococcus*	16S, V4	393	Canada	Use of reagent and amplification controls reported	Moossavi et al., 2019

Regarding interpretation of the results, surprising findings are sometimes not sufficiently scrutinized. For example, several studies on the hMM reported taxa such as *Bradyrhizobium*, a typical nitrogen-fixing soil bacteria (e.g., Cabrera-Rubio et al., 2012; Hunt et al., 2012; Li et al., 2017; Pannaraj et al., 2017). *Bradyrhizobium* has been highlighted as a signature contaminant in microbiome studies of samples with low bacterial abundance (Salter et al., 2014). Another unusual result was the report of a pronounced dominance of bifidobacteria detected by qPCR and complete absence of this taxon when evaluated by sequencing of the 16S rRNA gene (Cabrera-Rubio et al., 2012). Moreover, qPCR results indicated a 100-fold higher abundance of bifidobacteria (approx. 10^5^ CFU/ml) than most other studies (approximately 10^3^ CFU/ml) (Gronlund et al., 2007; Gueimonde et al., 2007; Collado et al., 2009; Martín et al., 2012; Khodayar-Pardo et al., 2014; Cabrera-Rubio et al., 2015) but the potential reasons for these differences were not explored. More detailed reporting of experimental details, including validation of sensitivity and specificity of the primer sequences is needed.

Despite these challenges, and many inconsistencies, a systematic review of studies exploring the hMM using culture-independent methods concluded that, regardless of geographic location or analytic methods, streptococci and staphylococci are the most predominant genera in human milk (Fitzstevens et al., 2017). Studies published since the Fitzstevens review are listed in Table 1 and support this conclusion. Interestingly, and in contrast to the gut microbiome, most taxa detected in HM should be amenable to culture using standard microbiological methods.

## Association Between Maternal Characteristics and the hMM Composition

A number of studies have associated several maternal characteristics, including delivery mode, stage of lactation, maternal BMI and diet, with hMM composition. Concerning the impact of the delivery mode, although six studies (average *n* = 49) reported some differences (Cabrera-Rubio et al., 2012, 2015; Kumar et al., 2016; Li et al., 2017; Toscano et al., 2017; Williams et al., 2017), none of the findings were consistent, and the five largest studies (average *n* = 152) found no differences (Soto et al., 2014; Sakwinska et al., 2016; Urbaniak et al., 2016a; Pannaraj et al., 2017; Moossavi et al., 2019). Regarding the impact of stage of lactation, four studies found no differences in bacterial composition or quantities assessed by qPCR (Collado et al., 2012; Sakwinska et al., 2016; Li et al., 2017; Pannaraj et al., 2017). The remaining three studies reported significant differences (Cabrera-Rubio et al., 2012; Khodayar-Pardo et al., 2014; Makino et al., 2015), but with poor consistency, except the common finding of a higher counts of bifidobacteria at later stage of lactation common to two of the studies (Khodayar-Pardo et al., 2014; Makino et al., 2015). The studies where no differences were observed had a larger sample size (Collado et al., 2012; Sakwinska et al., 2016; Li et al., 2017; Pannaraj et al., 2017) as compared to the studies where differences were found (Cabrera-Rubio et al., 2012; Khodayar-Pardo et al., 2014; Makino et al., 2015) (average *n* = 89 vs. *n* = 51). The association of maternal BMI with the hMM was evaluated in six studies, and although all studies reported some differences between normal and overweight mothers, none of these differences were consistent (Cabrera-Rubio et al., 2012; Collado et al., 2012; Dave et al., 2016; Kumar et al., 2016; Williams et al., 2017; Moossavi et al., 2019). Noteworthy, in a large study of Moossavi et al. (2019), the most prominent factor influencing hMM was the mode of breastfeeding (pumped vs. directly at the breast). However, as no other studies evaluated the influence of the breastfeeding mode on hMM composition, these results await replication, especially in the light of inconsistency of results among studies described above.

In conclusion, although individual studies reported significant associations of anthropometric or clinical variables such as the time of lactation, maternal BMI, and the delivery mode with the hMM composition, there was almost no consistency of findings across studies. Taken together with a trend for larger studies reporting no differences, we conclude that based on the existing evidence, there is no evident association between the maternal characteristics and the hMM composition.

## The Origin of Bacteria in Hm

How the bacteria reach human milk is not fully understood, and there appear to be discrepant views regarding the specific route to reach the mammary gland (Rodríguez, 2014). The mammary gland is open to the external environment and in the absence of specific and active mechanisms to maintain sterility, bacteria would dramatically infiltrate and grow. Live bacteria are present in the breast tissue of non-lactating non-pregnant women (up to 10^3^ cells/g tissue) (Urbaniak et al., 2014). This suggests that if they are actively transported, the process is not specific to pregnancy and lactation, but could increase in this period. For instance, the transfer of bacteria from the outside of the breast (either present on the skin or in the mouth of the baby) could be facilitated via retro-flow of milk into duct during suckling commonly called retrograde infant-to-mother transfer (Ramsay et al., 2004).

The concept of the enteromammary route was triggered by the observation that low numbers of live bacteria were present in the mammary gland of pregnant mice, and the maternal blood and milk cells contained bacterial DNA (Perez et al., 2007). Enteromammary transfer stipulates the existence of an active mechanism that transfers live bacteria from the maternal GI tract to the mammary gland via the mesenteric lymph node network (Perez et al., 2007; Rodríguez, 2014). A recent study of de Andres et al. (2017) documented translocation of two strains of lactococci, *L. lactis* and *L. salivarius* transformed with *luxABCD* and *luxAB* genes, respectively. The detection of *L. lactis* was purely qualitative using visualization of bioluminescent bacteria grown on selective plates. The detection of *L. salivarius* was carried out by specific PCR targeting *luxAB* gene fragments and, in parallel, observing bacterial growth on selective plates. Strikingly, the bacteria were detected in all investigated tissues (kidney, liver, and spleen) and body fluid (urine) that are normally free from bacteria. Thus, the translocation of bacteria does not appear unique to milk, raising questions about its specificity for the transport of live bacteria, and the ultimate role to provide a viable inoculum for the infant gut.

Bacterial translocation from GI tract constantly occurs at a low level and appears to be enhanced by a variety of pathological conditions associated with leaky intestinal barrier, such as metabolic syndrome (Brandsma et al., 2015) or autoimmune diseases (Mu et al., 2017). Even though poorly explored to date, the physiological changes during pregnancy and lactation share many similarities with these pathological conditions and are characterized by higher expression of inflammatory markers and alteration of gut barrier as shown in rodents, pigs, and humans (Smyth et al., 2005; Kerr et al., 2015; Cheng et al., 2018; Mokkala et al., 2018). Such transient gut barrier alteration may favor bacterial transfer from mother’s GI tract to other tissues.

Further details concerning the currently proposed innate-cell mediated selection of beneficial bacteria from maternal GI tract (Rodríguez, 2014) are not well documented beyond the description of unpublished data. The hypothesized existence of an active uptake mechanism presumably involving dendritic cells (DCs) suggests a potential benefit to infant. DCs should avoid GI pathogens and preferentially take up beneficial organisms, but it is not clear how such a mechanism could operate. This aspect is particularly important as the taxa considered beneficial to the neonate such as bifidobacteria, are not very abundant in the maternal GI tract due to the very different ecologies of the adult and neonatal gut. We also know that such DC/bacteria interactions even with non-pathogenic organisms should trigger DC phenotypic maturation which was not yet documented. The origin of the hMM is a challenging concept to carefully address experimentally. However, it is noteworthy that the active transport of bacteria to the mammary gland from maternal GI tract is neither the prerequisite for bacteria to be present in the HM, nor for their potential beneficial role in infant or maternal health.

## The Role of the hMM: Infant Gut Microbiome Seeding as the Predominant Hypothesis

Does the fact that healthy human milk contains small but usually detectable amounts of live bacteria imply their specific role in either maternal or infant health? Classical thinking would suggest that in the absence of other evidence and when there is no harm to either the mother or the neonate, the microbes represent a marginal contamination that would be costly and unnecessary for the body to entirely remove. While such a null hypothesis should not in our view be entirely neglected, the last decade has brought considerable number of studies that at least suggest a beneficial role of microbiomes for human health.

The predominant hypothesis regarding the hMM postulates that it plays a major role as a microbiome inoculum for the infant gut, an appealing concept named “mother nature’s prototypical probiotic food” (McGuire and McGuire, 2015). The key support for this hypothesis arises from comparisons of bacterial strains from the HM and infant feces, coupled with the notion that other routes of mother-to-infant transfer of live bacteria are difficult to envisage.

Several studies isolated bacterial strains from the HM and infant stool using the classical microbiology approach involving culturing bacterial strains and genotyping to establish their relatedness (Jiménez et al., 2010; Solis et al., 2010; Albesharat et al., 2011; Martín et al., 2012; Jost et al., 2014; Makino et al., 2015) and potential sharing. However, studies differed in the resolution of the genotyping methods used, and consequently the conclusions that can be drawn. Pulsed-field Gel Electrophoresis (PFGE), generally considered as gold standard for unambiguous genotyping was used in different reports (Martín et al., 2006, 2012; Jost et al., 2014). Others used methods with lower resolution, Random Amplified Polymorphic DNA (RAPD) (Solis et al., 2010; Albesharat et al., 2011), or Multilocus sequence typing (MLST) which is generally not considered suitable for strain identification (Makino et al., 2015). As bifidobacteria are often dominant in the infant gut microbiota and are considered beneficial, the majority of studies focused on the potential sharing of strains belonging to this genus. However, most studies found that only a minority of women harbored bifidobacteria in HM ranging from 2 to 11% (Solis et al., 2010; Martín et al., 2012; Jost et al., 2014; Soto et al., 2014). Makino et al. (2015) reported prevalence increasing from zero at delivery to 30% at 1 month post-partum. Interestingly, an early study (Martín et al., 2012) found evidence of more frequent sharing of *S. epidermidis* (50% of pairs) and *S. hominis* (25% of pairs) than of bifidobacteria (15% of pairs). Although reported sharing of lactobacilli strains in mother-infant dyad (Albesharat et al., 2011), the resolution of (RAPD) might have been too low to draw robust conclusions as the same RAPD genotype was found in multiple mother-infant dyads. Newer studies that attempted to address the strain sharing question using 16S rRNA gene amplicon sequencing (Biagi et al., 2017; Pannaraj et al., 2017) were challenged due to the inherently low resolution of this method, precluding strain-level resolution indispensable to answer questions about strain transfer. Shotgun metagenomics could in principle provide strain-level data, however, the only study that attempted to assess the role of the hMM in mother to infant bacterial transfer using this method reported that was not feasible due to too low bacterial DNA concentration precluding the generation of sequencing libraries (Ferretti et al., 2018). So far, the evidence from studies based on molecular detection methods appears limited.

In conclusion, there is evidence that identical strains could be found in infant feces and in HM, although in some studies the evidence is limited by low resolution of the utilized methods. Finding the identical and unique bacterial strains at two different sites implies transfer, but it does not resolve the question about its direction. Adult family members have been shown to share gut strains (Song et al., 2013) implying that close or intimate contact enables transfer of obligate anaerobic gut bacteria among individuals through sharing of a “personal bacterial cloud” (Meadow et al., 2015). It has been argued that mother and infant form an ecosystem where bi-directional passage of bacteria often occurs (Biagi et al., 2017) thanks to exceptional physical closeness. Indeed, careful longitudinal study reported that bifidobacteria strains identical to maternal stool isolates were without exception first found in infant stool before appearing in maternal milk (Makino et al., 2015). Aero-tolerant gut strains such as bifidobacteria could most likely be occasionally transferred from infant gut to HM.

Currently, the maternal GI tract is considered the most prominent source of bacteria for the infant gut, supported by recent shotgun metagenomics studies with stool samples from mother-infant dyads (Yassour et al., 2016; Asnicar et al., 2017; Ferretti et al., 2018; Korpela et al., 2018) as well as the earlier culture-based studies (Takahashi et al., 2010; Jost et al., 2014; Makino et al., 2015). Cesarean section has long been recognized as a factor associated with altered infant gut microbiota (Korpela and de Vos, 2018), leading to suggestion that maternal vaginal bacteria play a prominent role in inoculating the infant gut (Dominguez-Bello et al., 2016). Recent studies confirmed that Cesarean section leads to a severe disruption of maternal to infant microbial transfer, however, it is the inoculum from maternal GI tract rather than vagina that plays a predominant role in the seeding of the infant gut (Yassour et al., 2016; Korpela et al., 2018). This also implies that the relative role of the hMM in supplying the inoculum for the infant should be much greater in Cesarean section delivered infants.

One million bacteria are estimated to be ingested daily by breastfed infant, and this appears an impressive number. However, from the nearly complete absence of bacteria at delivery, the newborn is very rapidly colonized. Within a few days the number reaches approximately one trillion, and thus newly ingested bacteria have to display high competitiveness compared to the resident microbes. In the context of the seeding hypothesis, the potential role of the hMM in supplying inoculum to the infant gut should be the greatest within first few days of life. This does not preclude that the continued supply of microbes at later stages of lactation may affect some aspects of gut mucosal immunity.

Human milk is beyond doubt a key factor for shaping the infant gut microbiome through its known prebiotic and multiple bioactive properties (Hinde and Lewis, 2015). However, the specific role of the hMM in colonization or seeding of the neonatal gut remains, in our opinion, to be demonstrated. The focus on the seeding hypothesis may have been at the detriment of other investigations. The role of the dominant taxa of HM, staphylococci and streptococci, remain largely unexplored and only few studies have considered other potential roles of the hMM for maternal health.

## Beyond Nutrition: Evolutionary Origin of the Mammary Gland and Protective Functions of Hm

**FIGURE 1:**
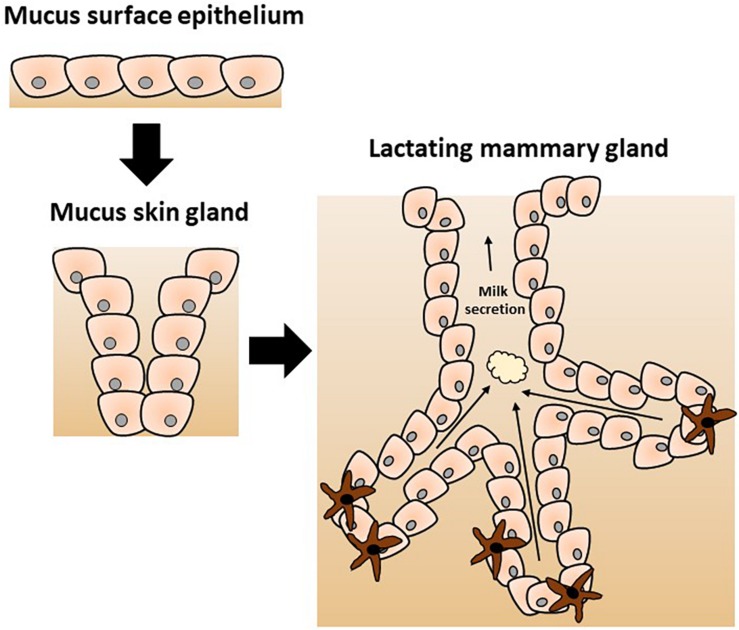
Proposed evolution of the mammary gland from a mucus-secreting epithelial skin gland adapted from Vorbach et al. (2006). The evolutionary model postulates the divergence of specific enzymes with additional functions as discussed in the text. The original immune protective role of the skin secretion was gradually modified to a nutritive role of the contemporary mammary gland with a new specific complex secretion product called milk.

Protective function has been proposed and convincingly argued to be the original role of the mammary gland. According to this hypothetical scenario, its more obvious nutritional role has evolved more recently, as originally proposed by Hayssen and Blackburn (1985), Oftedal (2002), and Vorbach et al. (2006) and elegantly reviewed by McClellan et al. (2008). In brief, a series of transitions from a mucus-secreting epithelium through a mucus skin gland and finally the contemporary mammary gland was proposed (as depicted in Figure 1). Hence, the mammary gland is hypothesized to have evolved from an epithelial barrier interface with protective function into a mammal-specific reproductive organ with protective and nutritional functions through lactation. Genetic and biochemical analysis of milk proteins showed that multiple molecules play a dual role in immunity and nutrition including lysozyme and α-lactalbumin as reviewed by Vorbach et al. (2006). Lysozyme is a known antimicrobial enzyme secreted by epithelium including lactating mammary epithelium. Lysozyme gene duplication gave rise to α-lactalbumin which is a major whey protein found in breast milk with direct nutritional properties. Of note, α-lactalbumin is also a key part of the lactose synthase enzymatic complex. Lactose is a major source of calories in breast milk; its other important function is to provide adequate viscosity for optimal suckling (Brodbeck et al., 1967; Vorbach et al., 2006).

As the mammary gland constitutes a secretory barrier surface with ancestral protective function, the presence of bacteria in mammary gland or breast milk should be explored in context of rapidly growing knowledge on the contribution of microbes to mucosal immunity. Most of the current knowledge is derived from studies of the GI tract and its impressive bacterial community. The GI mucosal immune system protects host tissues from harmful exposure to bacteria with a so-called inside-out control which requires (i) stratification and (ii) compartmentalization of the microbiota as reviewed in Gallo and Hooper (2012), Hooper et al. (2012), and Macpherson and McCoy (2013). Secretion of mucins, antimicrobial peptides or proteins (AMPs) and immunoglobulins (Ig) particularly IgA organizes stratification by minimizing direct contact of bacteria with the host epithelial cells and influencing microbiota composition (Bunker and Bendelac, 2018; Donaldson et al., 2018). Compartmentalization defines the local confinement, control, and killing of bacteria that breach the multiple epithelial barriers by a variety of immune cells particularly innate immune cells to limit their systemic spread (Shukla et al., 2017; Chen et al., 2018; Kroon et al., 2018; Cheng et al., 2019). The host-microbe symbiotic relationship also implies an outside-in influence exemplified by studies showing how the microbiota shapes host mucosal immunity (Smith et al., 2007; Macpherson and McCoy, 2013; Shukla et al., 2017; Chen et al., 2018; Kroon et al., 2018; Cheng et al., 2019). GF animals had impaired immunity including poor expression of some AMPs, significantly impaired specific and non-specific IgA levels or repertoire, and less populated and reduced lymphoid structures (Hooper et al., 2003; Smith et al., 2007; Vaishnava et al., 2008, 2011; Hansson et al., 2011; Gury-BenAri et al., 2016). Moreover, correction of the CD4^+^ T cells lymphopenia in GF mice can be obtained by colonization with *Bacteroides fragilis* through the production of Polysaccharide A (PSA) or purified PSA injection (Mazmanian et al., 2005). Some bacterial motifs exclusively released by gram-negative bacteria also induce the development of isolated lymphoid follicles (Bouskra et al., 2008). Likewise, bacterial metabolites such as short chain fatty acids (SCFAs) generated by carbohydrate fermentation are also important nutrients for epithelial cells and key signaling molecules with immunomodulatory properties (Meijer et al., 2010; Greer et al., 2013; Bolognini et al., 2016). In general, specific microbes and/or the associated compounds that may rectify GF associated alterations are still poorly defined.

Molecular attributes of stratification and compartmentalization are largely found in HM containing mucins, large amounts of AMPs and IgA as well as diverse immune cell types, which can evolve across lactation and respond to mother or infant infection (Schroten, 2001; Hassiotou et al., 2013b; Trend et al., 2015a; Donovan, 2019). This was illustrated by several biochemical and proteomic studies, as well as the more recent flow cytometry-based cell analysis of fresh HM samples across the world (Patton, 2001; Ballard and Morrow, 2013; Hassiotou et al., 2013a; Wada and Lonnerdal, 2014; Brunser, 2018). Hence, microbiota found in the resting mammary gland or HM might impact molecular and cellular processes behind stratification and compartmentalization resulting in a mucosal-like immune system organization in mammary tissue.

Epithelial cells (with several tissue-specific distinct lineages) produce the majority of AMPs within barrier surfaces, AMPs are also produced by a few infiltrating innate lymphoid cells (ILCs) (Britanova and Diefenbach, 2017). Expression, secretion and activity of AMPs are influenced by microbiota in transcriptional and post-translational stages as previously reviewed (Gallo and Hooper, 2012). Whereas the expression of some AMPs, for example lysozyme, is microbiota-independent, the transcriptional regulation of other AMPs is microbiota-dependent, including β-defensins (BD2), c-type lectins [such as regenerating islet-derived protein (REG)3γ] or ribonucleases [such as angiogenin 4 (ANG4)] (Hooper et al., 2003; Cash et al., 2006; Selleri et al., 2007; Abtin et al., 2008). Thus, expression of microbiota-dependent AMPs is largely reduced in GF mice as compared to conventional mice, whereas upon bacterial recolonization normal values are restored. The influence of the microbiota is also observed in post-translational events regulating the bioactivity of AMPs. Initially inactive propeptides need processing into bioactive AMPs and secretion into the mucosal lumen as described for cathelicidins, α-defensins and REG3γ with some contribution of microbial derived proteases (Ghosh et al., 2002; Yamasaki et al., 2006; Mukherjee et al., 2009; Schroeder et al., 2011). Most of our knowledge is limited to studies of classical mucosal interfaces, such as the GI tract, the influence of hMM on AMP expression and activity is as yet unknown but plausible. For instance, lactic acid bacteria found in breast milk contribute to the release of casein and whey protein derived AMPs, using proteolytic system of cell wall bound proteinases as previously reviewed (Mohanty et al., 2016). It has recently been suggested to classify the mammary gland as a mucosal organ, based on animal data (Betts et al., 2018), although the specific contribution of the milk microbiota was not addressed. We suggest to further explore the role of the microbiota in milk AMP synthesis, secretion and activity. Such studies should shed light on the role of the hMM in shaping mammary gland immunity.

## Beyond Nutrition: the Role of Lymphoid Cells in HM

Innate Lymphoid Cells were recently identified as tissue−resident innate lymphocytes. ILCs are mainly located at barrier surfaces, maintained and expanded locally instead of being continuously replaced with circulating hematopoietic progenitors. ILCs are instrumental in mucosal tissue homeostasis, morphogenesis, metabolism, regeneration, and growth (Britanova and Diefenbach, 2017). It was recently demonstrated that all three classes of currently defined ILCs were present in HM, based on signature transcription factors and cytokine expression. In HM, ILC type 1 outnumbered ILC type 3 and type 2 by 3 to 30 times, respectively (Baban et al., 2018). The presence of ILC in HM supports the notion that the mammary gland is a mucosal-like barrier tissue (Vorbach et al., 2006). More importantly, it also raises several questions. What is the contribution of ILC to shaping resting or lactating mammary gland immunity and associated microbiota? Breastfed babies reportedly swallow ∼20000 ILCs per mL of milk daily (Baban et al., 2018). Thus it is plausible that ingested ILCs and related-secretion products could contribute to neonatal immunity and gut microbiome.

While data are limited in humans (as reviewed in Moles et al., 2018), in rodent models, several groups have shown that oral transfer of breast milk maternal leukocytes (not only ILCs) can occur and reach Peyer patches, lung or thymus of suckling pups (Ghosh et al., 2002; Cabinian et al., 2016; Darby et al., 2019). Survival of breast milk maternal leukocytes in the GI tract is likely due to its immaturity with low stomach acidity, poor immune reaction to maternal antigens and low digestive enzyme expression (Torow et al., 2017; Zens et al., 2017; Lenfestey and Neu, 2018). Permeability of neonatal GI tract to maternal cells was already observed in the 1980s (Sheldrake and Husband, 1985). Recently, Darby et al. (2019) demonstrated that maternal cellular immunity against parasites can be transferred to suckling pups and persists into adulthood. This transgenerational long-lasting immunity is specifically mediated by maternal T-cell transfer via breast milk (Darby et al., 2019).

## The Role of Symbiotic Interactions in Mammary Gland Physiology

Bacteria reside in the resting mammary gland, as observed in studies conducted with human tissue biopsies from mammoplasty or reductive surgeries (Urbaniak et al., 2014, 2016b; Hieken et al., 2016). Therefore, mammary gland development and physiology during puberty, gestation and breastfeeding (usually multiple rounds), aging and disease (such as breast cancer) could be influenced by indigenous microbes as well as remote microbes, secreted motifs and/or metabolites. Cross-talk between the microbiota and gut stem cells was recently described in rodents, highlighting an unsuspected homeostatic contribution of the microbiota to GI tract physiology (Stedman et al., 2016). GI tract epithelial stem cells reside in crypts at the bottom of the villi in the GI tract, controlling their renewal. Crypt-specific core microbiota was identified in the caecum and colon crypts (Pedron et al., 2012). These results suggested that a particular set of bacteria provide selective advantage to the host. Cross-talk between crypt stem cells and bacteria was subsequently confirmed *in vitro* with organoid cultures or with mouse models (Neal et al., 2012; Nigro et al., 2014; Naito et al., 2017). Lgr5^+^ crypt stem cells express the pattern recognition receptors TLR4 and NOD2 to respectively sense lipopolysaccharides (LPS) or muramyl-dipeptide (MDP) bacterial motifs with opposing effects (death vs. cytoprotective, respectively) (Neal et al., 2012; Nigro et al., 2014; Naito et al., 2017). These findings in mice models deserve to be studied in man. Such interactions are likely to also occur in the mammary gland where mammary epithelial stem cells and even so-called breastmilk stem cells were characterized in animals (Dontu et al., 2003; Shackleton et al., 2006; Stingl et al., 2006) and humans (Hassiotou et al., 2012, 2013a). The HM stem cell potential to sense bacteria and associated response to a healthy hMM (yet to be defined) has to be studied. It could have substantial implications for physiology of the resting mammary gland, development of malignancy as well as successful lactation. Noteworthy, most of our knowledge on hMM came from milk analysis of healthy women. Therefore data on spatial structure and interactions between microbes and the epithelial interface of the mammary gland are lacking and deserve further studies as done with human GI tract (de Muinck et al., 2017; Seekatz et al., 2019).

## Symbiotic Interactions Shape Mothers’ Protection Against Infections

Control of infectious agents and regulation of the interactions with the microbiota are some hallmarks of mucosal surfaces and mucosal immunity. Below we will focus on the role of the hMM in protection against mothers’ infection, and in particular lactational mastitis. Lactational mastitis is a common infection of the lactating mammary gland with inflammation of the tissue. It is of high health relevance, as a major risk factor for the cessation of breastfeeding. The estimated prevalence vary widely, from 10 to 33% (Foxman et al., 2002; Angelopoulou et al., 2018). While milk stasis caused by poor suckling and inadequate breastfeeding practices is a well-defined risk factor, the etiology of mastitis is unclear. *S. aureus* is suggested as the main etiological agent, and to result in more severe infections (Osterman and Rahm, 2000). However, only approximately 30% of mastitis samples contained *S. aureus* (Kvist et al., 2008; Delgado et al., 2009; Mediano et al., 2017). The finding that most mastitis samples also contained *S. epidermidis* belonging to Coagulase-negative staphylococci (CoNS) led to a suggestion that it could be an under-reported etiological agent of mastitis (Delgado et al., 2011; Mediano et al., 2017). However, *S. epidermidis* is the most prevalent taxon found in milk of healthy women, and in a cross-sectional comparison CoNS were more prevalent in samples from healthy controls (90%), than in mastitis samples (83%) (Kvist et al., 2008).

The numerous cases of mastitis with no known etiology suggests that at least some cases may be caused by breakdown of the regulation of normally resident bacteria leading to overgrowth and/or modified activity of normally occurring bacterial taxa and augmented interaction with the host. The etiology of human mastitis is still poorly understood, especially the role of streptococci should be further explored. Toxin production by *S. aureus*, and its regulation by quorum sensing could also play a role but so far has not been explored in the context of human mastitis.

Staphylococci colonization is found at other barrier surfaces such as the anterior nares (*S. aureus*), skin (*S. epidermidis*) (Mulcahy and McLoughlin, 2016; Krismer et al., 2017; Nakatsuji et al., 2017) and upper respiratory tract (streptococci) (Bogaert et al., 2004). Understanding the transition of facultative pathogens from harmless colonization to a potentially life-threating invasive disease represents an important challenge and a knowledge gap that should be explored (Zipperer et al., 2016). Staphylococci numbers in the mammary gland are normally controlled via adequate expression of inducible AMPs like cathelicidins (LL-37) or β-defensins (hBD-2 and hBD-3) (Otto, 2010; Gallo and Hooper, 2012). Simultaneous examination of the microbial composition, with a focus on staphylococci, streptococci and AMP profiles (cathelicidins and β-defensins) in HM should be carried out. Otto recently reviewed the existing active skin AMPs against *S. aureus* and *S. epidermidis* (Otto, 2010). HM comprises high levels of some AMPs, for example lactoferrin, while others such as hBD1, hBD2, HD5 and LL-37 are present but at lower concentrations (Dawarkadas et al., 1991; Velona et al., 1999; Mehta and Petrova, 2011; Trend et al., 2015b, 2016).

If lactational mastitis is caused by a dysregulation of host microbiota interactions, the treatment with probiotic strains isolated from milk of healthy women as reviewed in Angelopoulou et al. (2018) could offer an attractive alternative to antibiotics. Two lactobacilli strains *L. fermentum* PS2 and *L. salivarius* CECT5713 (Arroyo et al., 2010; Fernandez et al., 2016; Hurtado et al., 2017) were examined in the context of both treatment and prevention of mastitis and have shown promising results, although more studies are needed (Amir et al., 2016). Little data exists on the underlying mode of action. Probiotics may possess direct or indirect antibacterial activity against *S. aureus*. It has been suggested that lactobacilli exert their function via two distinct mechanisms: by acidic pH shift, as well as secretion of a specific antimicrobial proteins effective against *S. aureus* (Kang et al., 2017). We would like to propose a third mechanism linked to the hMM-mediated regulation of host specific AMPs against *S. aureus* and possibly other bacterial taxa limiting transition from commensal colonization to infection. Some supportive evidence comes from a recent small clinical study exploring gene expression in epithelial cells isolated from HM obtained from women with mastitis before and after intervention with PS2 for 21 days (de Andres et al., 2018). Microarray data analysis revealed a significant upregulation of interferon response pathways as well as Dermcidin, a known skin AMP involved in host defense against *S. aureus* (Schittek et al., 2001; Otto, 2010). In conclusion, the emerging evidence suggests the importance of the hMM for shaping mother’s protection with mucosal-like immunity features.

## Conclusion

**FIGURE 2:**
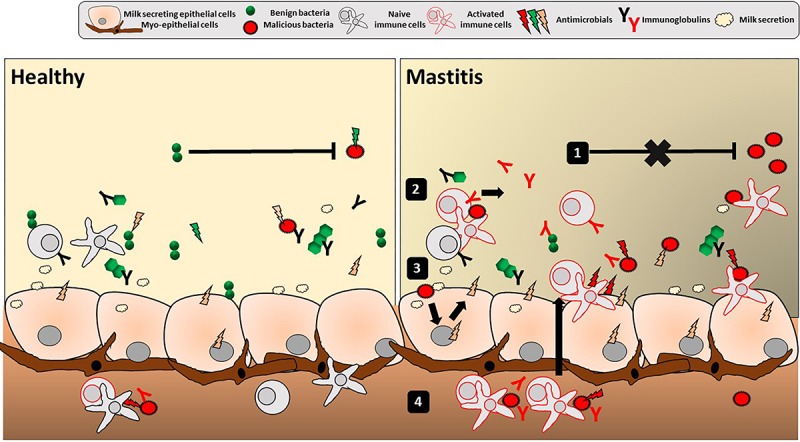
Mucosal immune features in the human mammary gland lobules. Depicted is the overall luminal interface of the mammary gland lobules and ducts where hMM cross-talk with epithelial cells occurs. Classical mucosal barrier immune features of stratification (with secretion of mucins, AMPs and IgA) and compartmentalization (with local confinement, control, and killing of bacteria) are present in breast gland and/or human milk, potentially playing a role in mother’s protection, mammary gland physiology and the hMM found under healthy conditions (left panel). Any alteration of stratification and compartmentalization could trigger transition from commensal colonization to detrimental hMM – host interactions and ultimately lead to mammary gland bacterial infection, i.e., mastitis (right panel). Altered hMM (1) may exert less bacteriostatic pressure against pathogens, (2) trigger non-specific inflammatory reaction and/or specific reaction (with immunoglobulins) against those pathogens as well as (3) epithelial cell reaction with secretion of proinflammatory mediators (cytokines, chemokines, and AMPs). Altogether, this leads to (4) attraction of additional activated immune cells by chemotaxis in the breast gland tissue and milk. The inflammation may result in breast pain, swelling, warmth, and redness characteristic to mastitis.

The hMM mostly consists of commensal staphylococci and streptococci, with inconsistent reports regarding other bacterial taxa, and no consistency regarding maternal and other variables influencing its composition. The methodological issues linked to working with low bacterial abundance samples are very likely an important source of these inconsistencies. The origin of the hMM has been explained by retrograde infant-to-mother transfer as well as the proposed existence of a so-called enteromammary route. hMM has been proposed to constitute initial inoculum for the infant gut, however, its significance remains to be demonstrated. The role of hMM in maternal health, linked to immune properties of the mammary gland, has been underexplored. We suggest that the epithelial organization and mammary gland secretion resembles many aspects of a mucosal barrier interface. Herein, the complex host-microbiota interactions could result in bacteriostatic or bactericidal activities shaping bacterial communities and immunity of the mother (as depicted in Figure 2), and possibly the immunity of neonates through breastfeeding. We also propose that quantitative and qualitative outcomes of lactation are likely to be dependent on these interactions. Thus, a reappraisal of the interactions between hMM and the mammary gland immune system should be carried out. It may have important clinical implications, particularly for mastitis research.

## Author Contributions

Both authors wrote and critically reviewed the manuscript.

## Conflict of Interest Statement

OS and NB are employees of Société des Produits Nestlé, SA.
